# The Application of Sulfur Influences Microbiome of Soybean Rhizosphere and Nutrient-Mobilizing Bacteria in Andosol

**DOI:** 10.3390/microorganisms11051193

**Published:** 2023-05-03

**Authors:** Jean Louise Cocson Damo, Takashi Shimizu, Hinako Sugiura, Saki Yamamoto, Shin-ichiro Agake, Julieta Anarna, Haruo Tanaka, Soh Sugihara, Shin Okazaki, Tadashi Yokoyama, Michiko Yasuda, Naoko Ohkama-Ohtsu

**Affiliations:** 1 United Graduate School of Agriculture, Tokyo University of Agriculture and Technology, Saiwaicho 3-5-8, Fuchu 183-8509, Tokyo, Japan; 2 National Institute of Molecular Biology and Biotechnology, University of the Philippines Los Baños, Los Baños 4031, Laguna, Philippines; 3 Faculty of Agriculture, Tokyo University of Agriculture and Technology, Saiwaicho 3-5-8, Fuchu 183-8509, Tokyo, Japan; 4 Graduate School of Agriculture, Tokyo University of Agriculture and Technology, Saiwaicho 3-5-8, Fuchu 183-8509, Tokyo, Japan; 5 Institute of Agriculture, Tokyo University of Agriculture and Technology, Saiwaicho 3-5-8, Fuchu 183-8505, Tokyo, Japan; 6 Faculty of Food and Agricultural Sciences, Fukushima University, Kanayagawa 1, Fukushima 960-1296, Fukushima, Japan; 7 Institute of Global Innovation Research, Tokyo University of Agriculture and Technology, Harumicho 3-8-1, Fuchu 183-8538, Tokyo, Japan

**Keywords:** arylsulfatase, malic acid, phosphate solubilization, siderophore, soybean, sulfur

## Abstract

This study aimed to determine the effect of sulfur (S) application on a root-associated microbial community resulting in a rhizosphere microbiome with better nutrient mobilizing capacity. Soybean plants were cultivated with or without S application, the organic acids secreted from the roots were compared. High-throughput sequencing of 16S rRNA was used to analyze the effect of S on microbial community structure of the soybean rhizosphere. Several plant growth-promoting bacteria (PGPB) isolated from the rhizosphere were identified that can be harnessed for crop productivity. The amount of malic acid secreted from the soybean roots was significantly induced by S application. According to the microbiota analysis, the relative abundance of *Polaromonas*, identified to have positive association with malic acid, and arylsulfatase-producing *Pseudomonas*, were increased in S-applied soil. *Burkholderia* sp. JSA5, obtained from S-applied soil, showed multiple nutrient-mobilizing traits among the isolates. In this study, S application affected the soybean rhizosphere bacterial community structure, suggesting the contribution of changing plant conditions such as in the increase in organic acid secretion. Not only the shift of the microbiota but also isolated strains from S-fertilized soil showed PGPB activity, as well as isolated bacteria that have the potential to be harnessed for crop productivity.

## 1. Introduction

Sulfur (S) is important in agricultural practices as it is in crop fertilization and component for plant defense against microbial pathogens. S requirement for optimal growth differs between 0.1% and 0.5% of the dry weight of plants [1]. Plants take up this nutrient in the form of sulfate (SO_4_^2−^) and assimilate it into cysteine and methionine, which are constituents for coenzymes and secondary plant metabolites. S plays significant roles in plant cell metabolic processes including redox reactions, synthesis of carbohydrates and lipids, detoxification of heavy metals, photosynthesis process, and plant response against abiotic and biotic stress [2,3,4,5]. As a component for biomolecules such as proteins, vitamins, and lipids, S is not only important for plants but also other living organisms such as microbes. Microorganisms take part in the biogeochemical cycle of S. In addition, S compounds can be utilized by microbes in the generation and conservation of its biochemical energy [6]; on that account, S is essential for the growth and development of living organisms.

The decline in industrial emissions of SO_2_ leads to depletion of SO_4_^2−^ in soil. As a result, the disturbance of S balance in the environment affects many countries such as Europe, North America, China, and India, where the average atmospheric S cannot meet crop nutritional requirements [7,8]. Moreover, S deficiency is typical in rural areas, particularly in humid tropics and temperate climates with highly leached soils [1]. Hence, the lack of S in soil for crop production is of great concern.

A soil’s fertilizer regime can play an important role in the acquisition of plant nutrients in the root system. The application of fertilizer granules with elemental S in durum wheat mobilizes the iron (Fe) in rhizosoil, thus providing more Fe to the crop [9]. Furthermore, S nutrition plays a vital role in the Fe uptake of barley. S deficiency in this crop leads to inhibition of Fe acquisition and accumulation in the shoot [10]. On the other hand, S application in soybean promotes organic acid production and increased phosphorus (P) availability in the rhizosphere [11]. It has also been observed that S fertilization may be involved in the induction of a citric acid transporter gene, *GmMATE13*, contributing to the excretion of the said organic acid in soybean roots [12]. Application of S in soybean may have significance on the P solubilization in the rhizosphere through augmentation of the organic acids produced in the roots.

In connection, the fertilizer regime also influences the soil microbial community [13,14], which can affect the nutrient acquisition of the plants; hence, the microbial component of nutrient mobilization, specifically P and Fe brought upon by S application, must be investigated. As it is affected by plant root activity, the narrow zone of soil adhered to roots referred to as the rhizosphere is a complex integrated network of plant roots, soil, and microorganisms [15,16]. Within the rhizosphere microbiome, plant growth-promoting bacteria (PGPB) is present, which can increase nutrient availability for plants, promoting their growth [17]. Direct mechanisms of PGPB include phosphate (P) solubilization, iron (Fe) sequestration, and arylsulfatase production. There are certain species of bacteria referred to as phosphate-solubilizing bacteria (PSB) that have the ability to mobilize bound phosphates into forms suitable for plant utilization. In return, the plants metabolize root-borne carbon compounds, mainly sugars and organic acids, for PSB growth [18,19,20]. In addition, microbe-mediated Fe uptake is one of the strategies plants use to obtain this essential nutrient from soil. PGPB can produce low molecular weight compounds known as siderophores that can chelate iron. Hence, siderophore-producing bacteria (SPB) can increase the availability of Fe for plant acquisition [21]. Lastly, PGPB can also synthesize and release arylsulfatase, which can hydrolyze sulfate esters into sulfate. Arylsulfatase-producing bacteria (APB) can extract S from soil organic matter, making it available for plants [22].

The living roots exude different types of substances or rhizodeposits, mainly consisting of carbohydrates, organic acids, secondary metabolites, and amino acids, and, consequently, shape the microbial community composition of the rhizosphere [23]. Root exudates are not vital only as a nutrient source for soil microbes but also act as signaling molecules in plant–microbe interactions [24]. For instance, the chemotactic nature of rhizobia towards the root exudates of legumes enables them to find their host legume plant at a distance [25]. Some legume exudates also induce symbiosis, such as daidzein and genistein for *Bradyrhizobium japonicum* and soybean [26] or the hyphal branching factor strigolactone for arbuscular mycorrhizal fungi and *Lotus japonicas* [27]. Root exudates thus actively influence the diverse microbial groups residing in the rhizosphere in a manner that can be beneficial, neutral, or detrimental to the plant. Indeed, knowledge on the root exudation of plants can be an opportunity to identify sustainable agricultural practices, specifically in the recruitment of plants to beneficial microbes.

Thus, this study aimed to determine the effect of S application on the rhizosphere microbial community in relation to root exudation, particularly organic acids, resulting in a rhizosphere microbiome with better nutrient mobilization capacity. To clarify the effect of S on the root-associated bacteria, high-throughput sequencing of 16S rRNA was used to analyze the microbial community structure of the soybean rhizosphere. In addition, the isolated bacteria from the soybean rhizosphere were tested for beneficial traits to enhance plant growth such as phosphate solubilization, siderophore production, and arylsulfatase activity. Lastly, several PGPB were identified that can be harnessed for nutrient mobilization of P, Fe, and S.

## 2. Materials and Methods

### 2.1. Soil Sampling and Analysis

A non-cultivated subsurface soil (20–30 cm soil depth) without fertilization was sampled at the Tokyo University of Agriculture and Technology (Tokyo, 35°68′ N and 139°48′ E, 65 AMSL). A portion of the soil was air dried and passed through a 2 mm sieve for soil analysis. Soil pH was determined using a 1:5 ratio of soil to deionized water [28]. The available S content in the soil filtrate with 0.8 N nitric acid was measured by using inductively coupled plasma mass spectrometry (Agilent 7800; Agilent Technologies, Santa Clara, CA, USA), as described in a previous study [29], using 2 ppb indium as an internal standard. In addition, soluble P content in the soil was quantified by preparing soil suspension with 0.002 M sulfuric acid followed by filtration. Then, the P content in the filtrate was determined by colorimetric analysis using the molybdenum sulfate method [30]. In addition to the S and P content [12], total carbon (C) and total nitrogen (N) were quantified with the dry combustion method using an NC analyzer (SUMIGRAPH NC TR22, Sumika Chemical Analysis Service, Ltd., Osaka, Japan) with glutamic acid as standard sample. Acid oxalate extractable Fe and aluminum (Al), which contain active Fe and Al oxides, aluminosilicates, and organically bound Fe and Al, were extracted using acidic ammonium oxalate solution (0.2M, pH 3.0) [31]. Dithionite–citrate-extractable Fe and Al, which contain free Fe and Al oxides, were also extracted using a sodium dithionite–citrate solution [32]. The Fe and Al concentrations in the extracts were measured using an atomic absorption spectrophotometer (Hitachi Z-5000 Series Polarization Zeeman, Hitachi Measuring Instruments Service Co., Ibaraki, Japan).

The soil used in this study was classified as Dystric Umbric Silandic Andosol [33], which is loamic, hyperhumic, and acidic, having a pH of 5.2 (Table S1). Total C content was 76.9 g C kg^−1^ while 4.9 g N kg^−1^ was estimated for total N content. As described by Sugiura et al. 2022, the test soil was deficient to both available S (21.3 mg S kg^−1^) and soluble P (2.3 mg P_2_O_5_ 100 g^−1^) with high P adsorption capacity (2756.6 mg P_2_O_5_ 100 g^−1^). Amorphous Fe_o_ (29.2 g kg^−1^) and Al_o_ (71.1 g kg^−1^) from the test soil were extracted using the acid oxalate method. The Fe and Al extracted from dithionite–citrate reduction method were Fe_d_ (43.6 g kg^−1^) and Al_d_ (17.4 g kg^−1^).

### 2.2. Soybean Pot Experiment

The collected soil was used for pot experiment assay with non-S (NS) and S applications. The soil was added with chemical reagents as nutrients. As for S sources, K_2_SO_4_ and MgSO_4_•7H_2_O were amended in the S-application (Table S2).

*Glycine max* (L.) Merr. “Enrei” seeds were surface sterilized with 70% (*v*/*v*) ethanol and 3% (*v*/*v*) sodium hypochlorite and washed thoroughly with sterile distilled water. Then, the seeds were sown in the prepared 330 g of soil in a pot (outer diameter: 10.5 cm × length: 9 cm) with sterile distilled water at 60% of water holding capacity of the soil (0.66 g g ^−1^). The soybean plants were grown in a growth chamber room under controlled environmental conditions of 25 °C with 16 h light (300 μmol^−1^ m^−2^ s^−1^) and 8 h dark cycles for 6 weeks. The experiment was carried by using five replicates per treatment with each pot containing two plants. Six weeks after sowing, the samples were harvested. After removal of loosely adhered soil, the root zone was carefully placed in a bag for rhizosphere collection. The strongly adhered soils affected by the soybean roots were shaken off vigorously and used as rhizosphere soil samples [34]. The collected soils were stored at 4 °C for culture-dependent study and at −30 °C for amplicon analysis. For the plant samples, the separated shoots and roots were oven dried at 80 °C for 48 h to determine the dry weight and subsequently used for quantification of plant P content.

### 2.3. Plant P Content and Organic Acid Secretion from Roots

The aboveground and root parts of the soybean dried at 80 °C for 48 h were separately powdered using a grinder. The powdered samples were then acid digested using HNO_3_. The P concentration was quantified using the molybdate blue method [30] with four replicates.

Organic acids may have a role as chelating agents in mobilization of plant nutrients and shape the microbial community in the rhizosphere. Thus, the influence of S application on organic acid secretion using soybean roots was assessed. Before oven drying, the harvested roots of 6 week after sowing soybean plants were gently washed with tap water and rinsed with milli-Q water. The roots were soaked in 100 mL milli-Q water for 2 h at room temperature in order to collect the root exudates containing organic acids; then, the extracted solution was freeze dried. The lyophilized samples were redissolved with 2 mL milli-Q water and filter sterilized with a 0.22 µm membrane (Millex^®^-GV, Millipore, Cork, Ireland). The organic acids were analyzed using a high-performance liquid chromatography system equipped with Shim-pack column SCR102H and RSpack KC-811 (Shimadzu Co., Kyoto, Japan) at a column temperature of 40 °C, and the UV detector SPD-20A (Shimadzu Co., Kyoto, Japan) at 210 nm [12]. Moreover, the mobile phase used was 0.1% H_3_PO_4_ with a flow rate of 0.5 mL min^−1^ and measurement time of 50 min. The standards used in the analysis were malonic, malic, tartaric, and citric acids. The analysis was carried out using five replicates.

### 2.4. DNA Extraction and Amplicon Analysis

High-throughput sequencing of 16S rRNA was performed in NS- and S-treated bulk and rhizosphere soils. DNA extraction from 0.5 g of soils stored at −30 °C was performed using ISOIL for bead beating (NIPPON Genetics Co., Ltd., Tokyo, Japan) according to the manufacturer’s protocol. DNA quality and quantity check were conducted by using NanoDrop 1000 (Thermo Fisher Scientific, Waltham, MA, USA) and stored at −30 °C.

Amplification of V3/V4 regions of the bacteria were carried out by using PCR primers V3V4f_MIX and V3V4r_MIX (Bioengineering Lab. Co., Sagamihara, Japan). The first PCR was performed using KOD-Plus-Neo enzyme (Toyobo Co., Ltd., Osaka, Japan) in a final PCR volume of 20 µL with 100 ng of DNA. The PCR reaction was performed in a Simpliamp thermal cycler (Thermo Fisher Scientific, Waltham, MA, USA). Then, the PCR products were purified using FastGene Gel/PCR Extraction Kit (NIPPON Genetics Co., Ltd., Tokyo, Japan). Consequently, tailed PCR was conducted using 2 µL of PCR product according to the sequencing company’s instructions. Then, purification of PCR product was performed using the same kit as in the 1st PCR reaction. Quantification of the PCR products was performed using Synergy H1 (BioTek, Winooski, VT, USA) and the QuantiFlour dsDNA system. Fragment analyzer and dsDNA 915 reagent kit (Advanced Analytical Technologies, Inc., Ankeny, IA, USA) were used to evaluate the quality of the libraries. Sequencing analysis was performed using MiSeq system and Miseq Reagent Kit ver 3.0 (Illumina, San Diego, CA, USA). The raw high throughput sequencing data were then processed in Quantitative Insights into Microbial Ecology (QIIME2.0). Lastly, the processed sequencing data were analyzed in R software Ampvis2 package and MicrobiomeAnalyst [35].

### 2.5. Isolation of Microorganism

The rhizosphere soil (10 g) was suspended in 90 mL 0.85% (*w*/*v*) NaCl solution using an orbital shaker (120 rpm) for 1 h. The serially diluted suspensions were plated on National Botanical Research Institute P (NBRIP) medium [36], modified Reyes’ medium [37], Luria-Bertani (LB) with chrome azurol-S (CAS) (Sigma-Aldrich, Co. Llc, St. Louis, MO, USA) reagent [38], and M9 minimal medium [39] to quantify cultivable bacteria. NBRIP medium was supplemented with 5 g L^–1^ Ca_3_(PO_4_)_2_ (Ca-P) (FUJIFILM Wako Pure Chemical Co., Osaka, Japan) as the sole insoluble P source while the Reyes’ medium was supplemented with AlPO_4_ (Al-P) (Kanto Chemical Co., Inc., Tokyo, Japan). Both media were used to isolate phosphate-solubilizing bacteria (PSB), specifically Ca-PSB for the former and Al-PSB for the latter, as observed with halo zones around the colonies after 7 days of inoculation. Siderophore-producing bacteria (SPB) were cultivated using LB medium with CAS reagent. Colonies of SPB showed yellow to red halo zones after 48 h. Lastly, the chromogenic arylsulfatase substrate 100 mg L^−1^ of 5-bromo-4-chloro-3-indolyl sulfate (Sigma-Aldrich, Co. Llc, St. Louis, MO, USA) was incorporated to the M9 minimal media for arylsulfatase-producing bacteria (APB). Bacteria with arylsulfatase activity were observed as blue colonies on the medium after 48 h. Colonies having halo zones or blue in color at 1/1000 dilution were chosen as isolates. Single colonies were selected, re-cultured in tryptic soy (TS) medium for PSB (30 isolates), LB medium for SPB (8 isolates), and M9 minimal medium for APB (12 isolates) and maintained as 40% (*v*/*v*) glycerol stocks at –80 °C until needed. Colony and cellular morphologies were observed in the selected isolates. Gram reaction using Gregersen’s method was also performed [40].

### 2.6. PCR Amplification of 16S rRNA Gene Sequence and Phylogenetic Analysis

In total, fifty bacterial isolates were identified using PCR amplification and sequence analysis of 16S rRNA gene. The isolates were grown in their respective media at 28 °C for 24 h. The cells were harvested and washed with sterile distilled water. Then, genomic DNA extraction was carried out using a Wizard^®^ Genomic DNA Purification Kit (Promega Co., Fitchburg, WI, USA) according to the manufacturer’s protocol.

Polymerase chain reaction (PCR) and sequencing of the 16S rRNA gene were performed using two sets of primers 1F/3R [41] and 800F/926R [42]. The gene was amplified using 10 ng of purified DNA and KOD Plus Neo (Toyobo Co., Ltd., Osaka, Japan). The PCR products were purified using a FastGene™ Gel/PCR Extraction Kit (Nippon Genetics Co., Ltd., Tokyo, Japan) and then the purified products were sequenced using an ABI PRISM 3500 Genetic Analyzer (Applied Biosystems, Waltham, MA, USA), following the manufacturer’s instructions. The obtained sequences were compared to 16S rRNA sequences deposited in the GenBank database using the BLAST online software (http://www.ncbi.nlm.nih.gov/BLAST, accessed on 16 July 2021). Phylogenetic trees based on the nucleotide sequences of the 16S rRNA gene sequences were aligned using Genetyx version 11 (Genetics Co., Ltd., Tokyo, Japan). The phylogenetic trees were constructed based on the neighbor-joining algorithm with 1000 replications using the bootstrap method and the maximum composite likelihood model without topology. These processes were carried out using Molecular Evolutionary Genetics Analysis (MEGA) software (version 6.0; Pennsylvania State University, State College, PA, USA) [43].

### 2.7. Plant Growth-Promoting (PGP) Characteristics

#### 2.7.1. Phosphate-Solubilizing Bacteria (PSB)

Twenty isolates were tested for their ability to solubilize P from Ca-P in NBRIP. On the other hand, ten bacteria were evaluated in Reyes’ medium with Al-P. PSB were grown in TS broth at 28 °C for 24 h; then, the bacterial cells were harvested by centrifugation at 10,000× *g* for 15 min. The cells were resuspended with 0.85% (*w*/*v*) NaCl at cell density of 10^7^ colony-forming unit per mL (CFU mL^−1^). Ten microliters of resuspended bacterial cells were spotted on the P growth media. There were three replicates of each isolate type. The plates were incubated for 7 days at 30 °C in the dark. The halo zone formation around the bacterial colony indicated that the isolate was able to solubilize phosphate. The phosphate-solubilizing ability of each isolate was evaluated by measuring the size of the halo zone, and the phosphate solubilization index was calculated as described previously [44].

#### 2.7.2. Siderophore-Producing Bacteria (SPB)

Eight SPB were grown in LB medium at 28 °C for 24 h. The bacterial cells were then harvested by centrifuge at 10,000× *g* for 15 min and resuspended with 0.85% (*w*/*v*) NaCl at a cell density of 10^7^ CFU mL^−1^. The resuspended cells (10 µL) were drop plated on LB medium with CAS reagent. The plates were incubated for 3 days at 30 °C in the dark. The siderophore production ability of the isolates were observed by yellow or orange halo zones. The index was calculated by dividing the total (including halo) diameter with the colony diameter.

#### 2.7.3. Arylsulfatase-Producing Bacteria (APB)

Twelve APB grown in M9 minimal medium at 28 °C for 24 h were estimated for their arylsulfatase production activity. Bacterial cells were harvested by centrifugation and resuspended with 0.85% (*w*/*v*) NaCl at cell density of 10^7^ CFU mL^−1^. Ten microliters bacterial resuspension were transferred in M9 minimal medium with chromogenic arylsulfatase substrate and incubated for 3 days at 30 °C in the dark. The activity was qualitatively estimated by the intensity of blue color in the colonies (Figure S1).

#### 2.7.4. Multiple Nutrient Mobilization Trait Screening

From the initial 50 bacteria, fifteen isolates (4 Ca-PSB, 4 Al-PSB, 4 SPB, and 3 APB) were selected based on their respective PGP abilities. These isolates were used to determine isolate that has multiple capabilities for PGP traits on nutrient mobilization. Bacterial resuspensions (10 µL) with cell density of 10^7^ CFU mL^−1^ were spotted on NBRIP, Reyes’, LB with CAS reagent, and M9 minimal with 5-bromo-4-chloro-3-indolyl sulfate media. Indices were computed from the halo zones produced on P growth media and LB with CAS reagent. The intensity of blue color on colonies were used for evaluation of arylsulfatase production of the isolates.

### 2.8. Statistical Analyses

Pot experiment data were statistically analyzed using IBM SPSS (v. 23.0.0; SPSS, IBM, Armonk, NY, USA). The amplicon sequence data were analyzed using MicrobiomeAnalyst. The microbial abundance (Chao1) and diversity (Shanon and Simpson indices) were determined using univariate analysis. Principal coordinate analysis (PCoA) based on the Bray–Curtis distance method was also carried out to visualize the clusters of the bacterial community and PERMANOVA to determine the effects of soybean cultivation and S application on the microbial community structure. To further evaluate the impact of S-application on soybean microbiome, linear discriminant analysis effect size (LefSe) assessments were carried out in rhizosphere soils specifically. Redundancy analysis (RDA) was performed to assess the relationship between the organic acids excreted by the soybean with NS and S conditions. Spearman’s rank correlation and Tukey’s test were carried out using SAS software (SAS Institute Inc., Cary, NC, USA). Lastly, principal component analysis was visualized using Genstat (VSN International, Hemel Hempstead, UK).

### 2.9. Nucleotide Sequence Accession Numbers

The DNA sequences were deposited in the DNA Data Bank of Japan (DDBJ) under accession numbers LC682200 to LC682249 (16S rRNA). The raw sequencing reads from the amplicon analysis were submitted to DRA/DDBJ under accession number DRA013637.

## 3. Results

### 3.1. Influence of S Application on Soybean and Its Microbial Community

**Figure 1:**
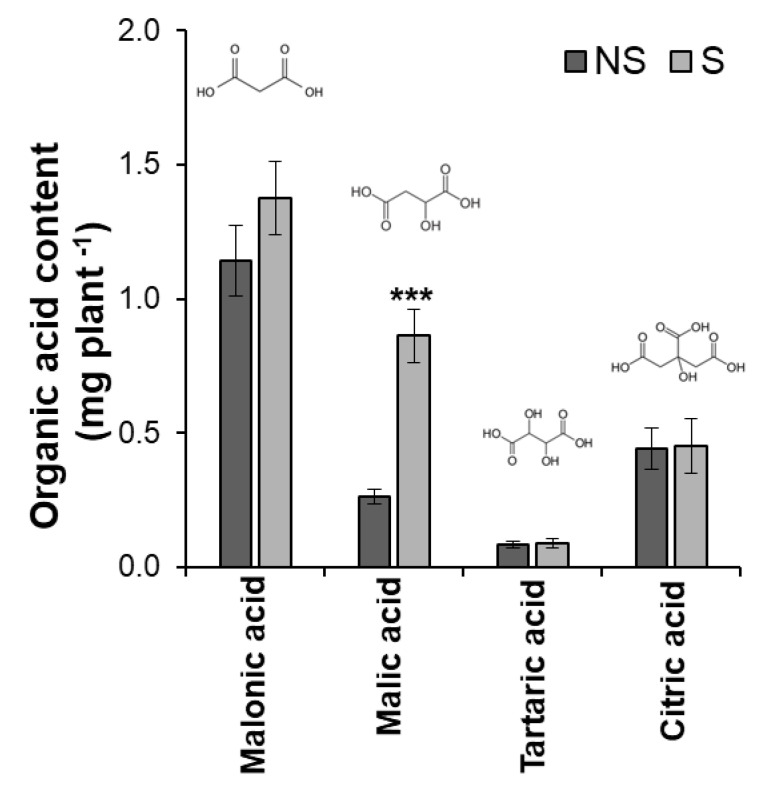
Organic acid content secreted from the roots of soybean cultivar Enrei six weeks after sowing under non-sulfur (NS) and sulfur (S) applications. Significant differences between the treatments were determined using the *t*-test (*** *p* < 0.001). The error bar indicates the standard error of four replications.

To determine the effect of S application on the microbial community structure in bulk soils and rhizosphere of soybean, pot experiments were conducted in S-deficient soil with/without S application. Although S application did not greatly affect the growth and P content of six-week-old plants (Figures S2 and S3), the amount of secreted malic acid from the roots significantly increased approximately threefold in the S-applied (S) soybean compared to the non-S (NS)-applied soybean (Figure 1).

Amplicon analysis was utilized to evaluate the influence of S application on the microbiome. A total of 282,666 16S rRNA sequences were obtained with an average of 23,555 read count per sample (range:18,967 to 34,579) from the soil and rhizosphere samples. The reads were clustered into 1902 microbial OTUs. The rarefaction curve reached saturation at ~7000 sequences and the species richness was higher in bulk soil fertilized with S (Figure S4).

### 3.2. Soil Microbial Diversity When Affected Using Soybean Cultivation and S Application

**Figure 2:**
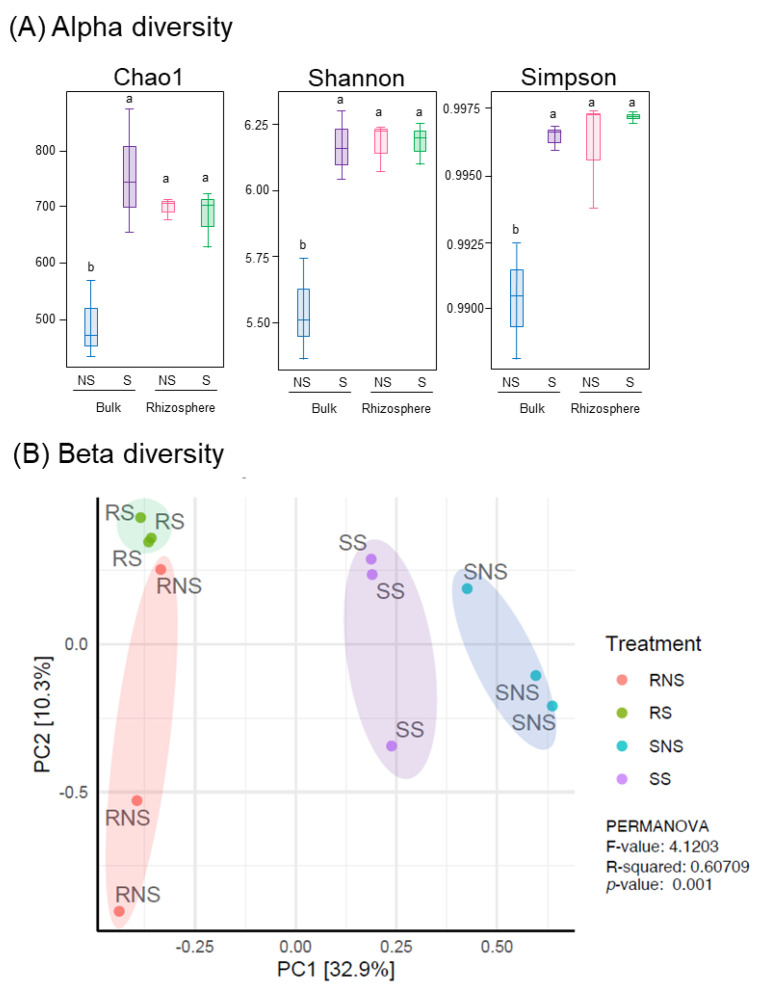
Influence of S application on the microbiota of the soil and the soybean rhizospheres. Measurement of the alpha diversity (**A**) and beta diversity (**B**) of the soil and soybean root rhizosphere with- or without S application. (**A**) Chao1, Shannon, and Simpson indices were used in the alpha diversity analysis. All values are means of three replicates ± standard errors. Statistical analysis was performed; different letters indicate significant difference between treatments (Student–Newman–Keuls (SNK) test, *p* < 0.05, *n* = 3). (**B**) Principal coordinate analysis (PCoA) were plotted based on Bray–Curtis distance metrices for taxonomical data (*p* < 0.001). Permutational multivariate analysis of variance (PERMANOVA) was performed.

The α-diversity of the bacterial community was evaluated based on richness (Chao1) and diversity (Shannon and Simpson indices) (Figure 2A). In bulk soil samples, S application brought significantly higher microbial abundance and diversity compared to NS. On the other hand, the microbial composition in rhizosphere soils was not altered substantially, regardless of S application. PCoA indicated the clustering of the bacterial community and PERMANOVA revealed that there was a significant shift in microbial structure with soybean cultivation (*p* < 0.001) (Figure 2B; Table S3). Moreover, the bacterial communities had a tendency to congregate based on the application of S.

The soil microbial community composition was visualized based on cultivation and S application at phylum level (Figure S5). Across the samples, the dominant phyla were Proteobacteria and Acidobacteria (Figure S5A). Irrespective of S application, the phyla Euryachaeota, Chloroflexi, Nitrospirae, and Acidobacteria had a tendency to increase with soybean cultivation (Enrei) (Figure S5B). In contrast, the abundance of phyla Proteobacteria, Bacteroidetes, and Actinobacteria reduced with planting of soybean. In the same manner, S application had lesser abundance for Bacteroidetes, Proteobacteria, and Actinobacteria, regardless of soybean cultivation (Figure S5C). Lastly, the phylum Acidobacteria had a higher quantity in S than in NS without considering the planting of soybean.

**Figure 3:**
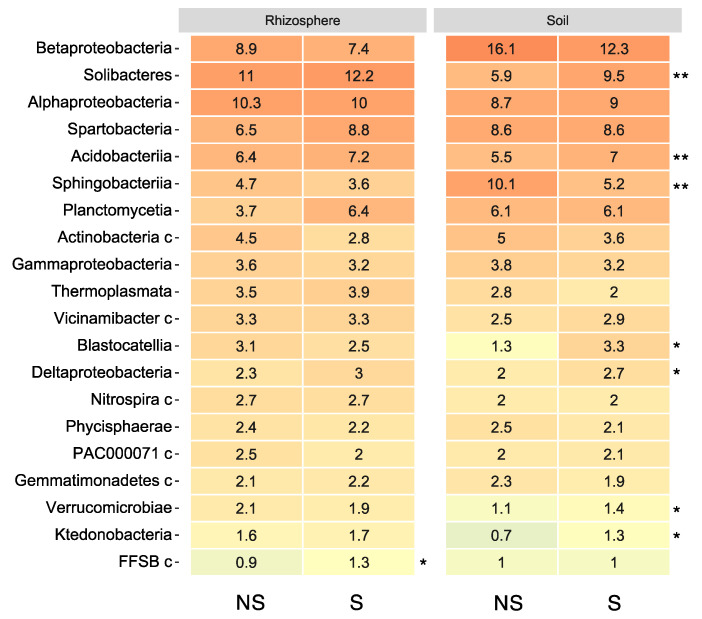
Heatmap of the top 20 bacterial classes associated with soil and soybean root rhizosphere with/without S application. The heatmaps show the relative abundances of bacterial operational taxonomic units (OTUs) at the class level from soil and soybean rhizospheres with or without S application. The color of the heatmap indicates the relative abundance from high (red) to low (blue). The asterisks identify significant differences between S-applicated (S) and non-S-applicated (NS) soils analyzed using the unpaired two-tailed Student’s t-test (* *p* < 0.05, ** *p* < 0.01).

In addition, the relative abundance of the top 20 bacterial classes was presented using a heatmap (Figure 3). Betaproteobacteria were prevalent in bulk soils while Solibacteres were prevalent in the rhizosphere. After S application in bulk soil, the relative abundance of some bacteria such as in Solibacteres, Acidobacteriia, and Deltaproteobacteria was significantly increased. In contrast, the abundance of Sphingobacteriia decreased with S application in bulk soils.

### 3.3. Soybean Rhizosphere Microbial Community Composition between S Applications

**Figure 4:**
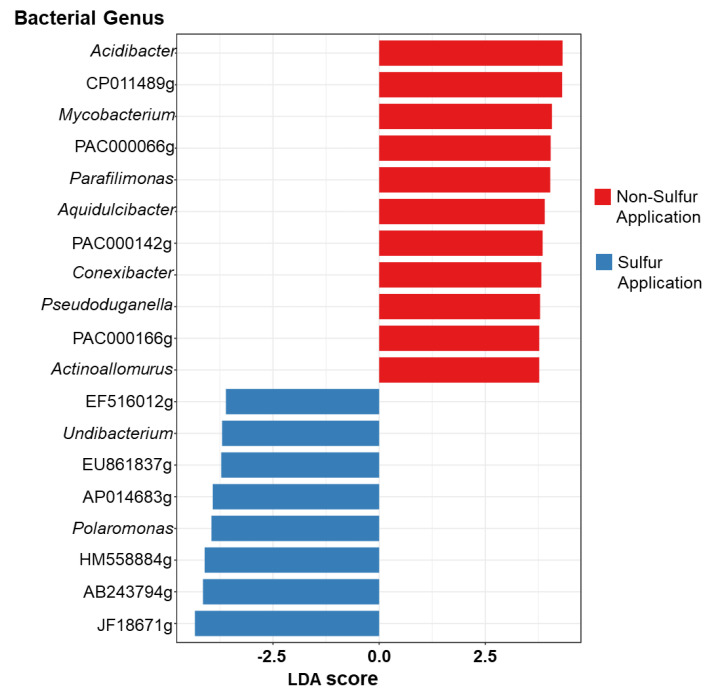
Linear discriminate of the effect size (LEfSe) of the preferential bacterial genus under S applications in soybean rhizosphere.

To further evaluate the impact of S application on the soybean microbiome, linear discriminant analysis effect size (LEfSe) assessments were carried out in rhizosphere soils specifically (Figure 4). LEfSe analysis (LDA score > 3.6) revealed that the NS-fertilized rhizosphere had a higher number of differential bacterial genera than the S-applicated rhizosphere. The genus *Acidibacter* from NS had the highest LDA score of 4.32. A high portion of genera favored in NS were classified under Actinobacteria such as *Mycobacterium*, *Conexibacter*, and *Actinoallomurus.* Moreover, Proteobacteria such as *Aquidulcibacter*, *Pseudoduganella*, and *Parafilimonas* (Bacteroidetes) were classified under NS. On the other hand, bacterial genera under Proteobacteria, namely *Undibacterium* and *Polaromonas*, were preferential in S application.

**Figure 5:**
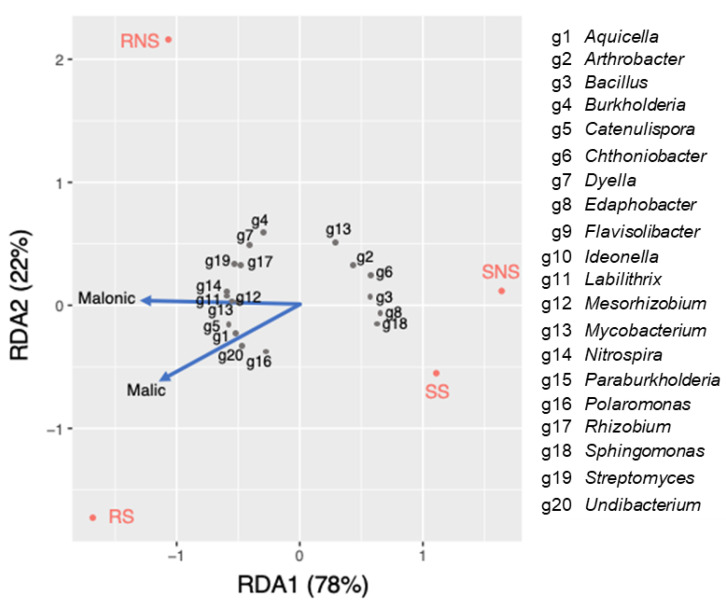
Redundancy analysis plot of the correlation between selected bacterial genera and organic acids excreted from soybean plants under NS and S application.

Redundancy analysis (RDA) was performed to assess the relationship between the bacterial genera and organic acids excreted by the soybean with NS and S (Figure 5). Spearman’s rank correlation was also performed to evaluate the significance of the relationships (Table S4). The first two RDA (RDA1 and RDA2) components explained 78% and 22% of total variance in the microbial community, respectively. It was found out that *Catenulispora* (ρ = 0.64), *Aquicella* (ρ = 0.57), *Undibacterium* (ρ = 0.78), and *Polaromonas* (ρ = 0.52) exhibited a positive relationship with malic acid in the sulfur-applied rhizosphere. In contrast, the abundance of *Mycobacterium* (ρ = −0.54) and *Arthrobacter* (ρ = −0.53) had negative correlation with malic acid. Furthermore, *Mesorhizobium* (ρ = 0.82), *Nitrospirae* (ρ = 0.59), and *Paraburkholderia* (ρ = 0.80) showed positive associations with malonic acid.

### 3.4. Quantification of Cultivable Nutrient-Mobilizing Bacteria in the Rhizospheres of NS- and S-Applied Soybean

The percentage (over the total nutrient mobilizing bacterial population) of bacteria, with their respective activity, is shown in Figure S6. The percentage of population count of Ca-PSB and APB were not distinct in NS and S. The presence of bacteria with siderophore activity was 1.8% in S while it was as high as 3.0% in NS. There was a tendency for SPB to be more abundant in NS- compared to S-applied rhizosphere soils. Lastly, the occurrence of bacteria that dissolved aluminum phosphate (Al-PSB) was 22.2% in S compared to 19.9% in NS.

The relationships between the plant traits (P content and organic acids) and population of the isolated nutrient-mobilizing bacteria were visualized in an RDA plot (Figure S7). The two RDA axes explained 87% of the total variance in the nutrient-mobilizing bacterial community. It was noted that the shoot’s P content and malic acid exhibited positive relationships. Furthermore, the populations of siderophore producers were associated positively with citric acid while being in negative relation with malic acid. An abundance of arylsulfatase-producing bacteria showed positive relations with tartaric acid.

### 3.5. Diversity in Nutrient-Mobilizing Bacteria Isolated from NS- and S-fertilized Soybean

A total of fifty isolates were selected for 16S rRNA gene sequencing and plant growth-promoting (PGP) screening. With equal number of isolates regarding treatment, 20 Ca-PSB isolates, 10 isolates with aluminum phosphate solubilization ability, 8 bacteria that are siderophore producers, and 12 arylsulfatase-producing bacteria were used for further analysis. The characteristics and identity of the isolates are summarized in Table S5.

**Figure 6:**
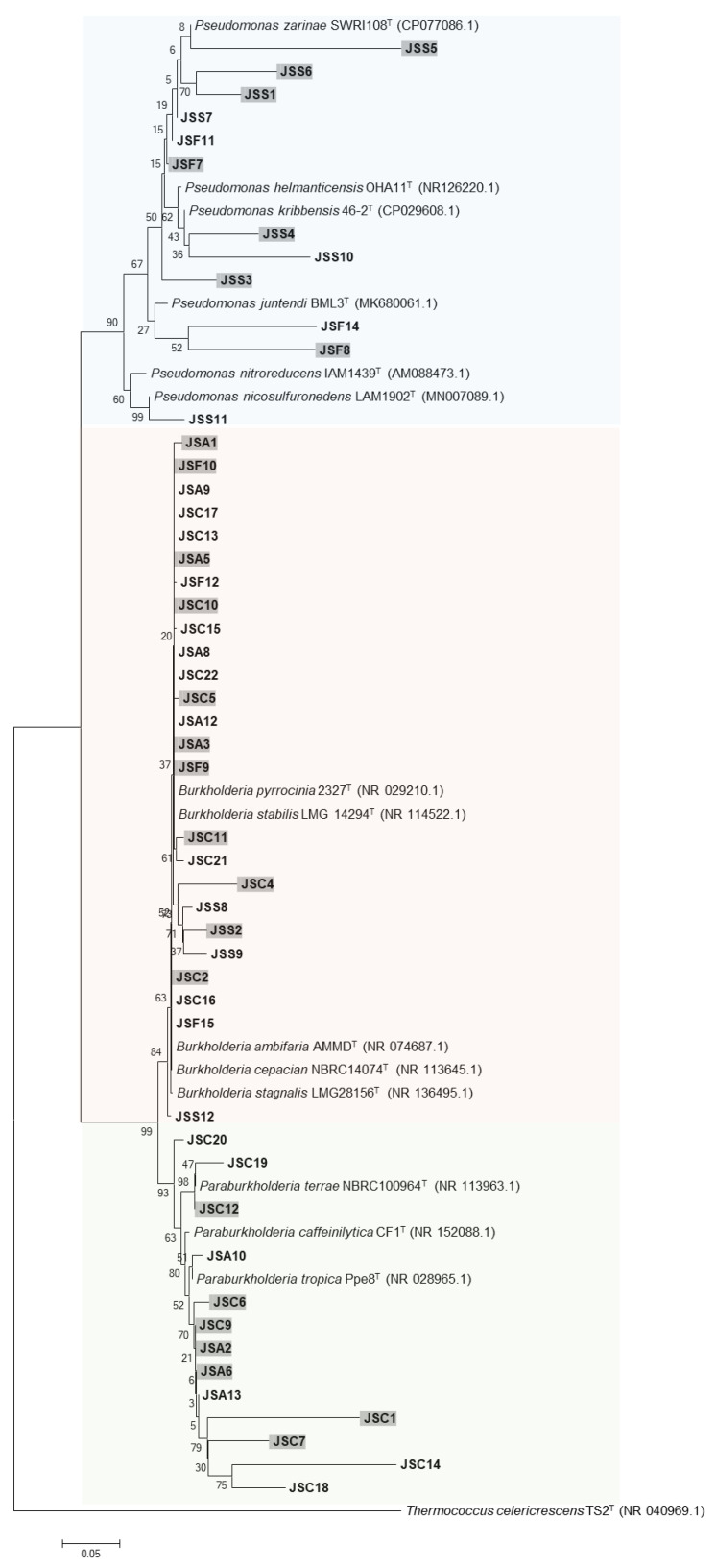
Phylogenetic tree based on 16S rRNA gene sequencing of isolates from non-sulfur- and sulfur-applied soybean rhizospheres and type strains. Bacteria isolated from soybean rhizosphere applied with S are highlighted in gray. Numbers at the nodes indicate the level of bootstrap support (%) based on a 1500 bp DNA fragment and neighbor-joining analysis with 1000 replications. The scale bar indicates 0.05 changes per site.

**Figure 7:**
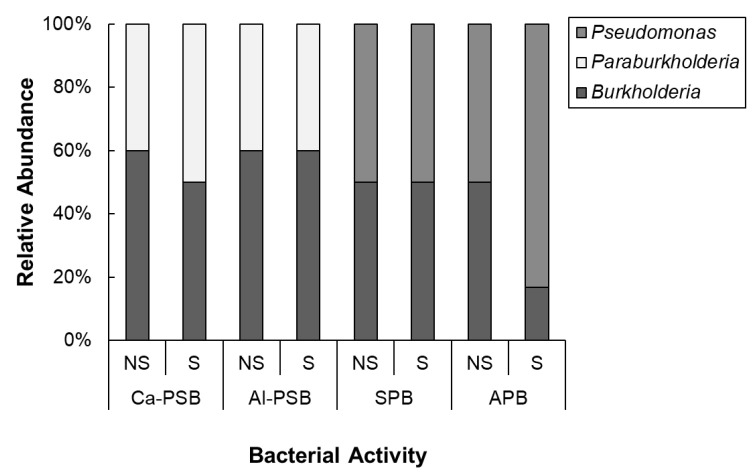
Relative abundance of the isolates regarding the activity and S applications at the genus level. NS: non-sulfur application; S: sulfur application; Ca-PSB: bacteria that can solubilize phosphorus from tricalcium phosphate; Al-PSB: bacteria that can solubilize phosphorus from aluminum phosphate; SPB: siderophore-producing bacteria; APB: arylsulfatase-producing bacteria. The identity of the isolates was based on 16S rRNA gene sequencing.

The phylogenetic relationships among the isolates grouped in S were strengthened by using an outgroup (*Thermococcus celericrescens* TS2), as shown in Figure 6. Phylogenetic analysis was used to classify the isolates into *β-Proteobacteria* (76%) and *γ-Proteobacteria* (24%). At genus level, the bacteria were categorized as *Burkholderia* (25 isolates), *Paraburkholderia* (13 isolates), and *Pseudomonas* (12 isolates). The relative abundance at genus level in consideration of bacterial activity and S application is presented in Figure 7. Regardless of the bacterial activity and fertilization, occurrence of *Burkholderia* was observed. The genus *Paraburkholderia* was observed only in phosphate solubilizers (Ca-PSB and Al-PSB) while *Pseudomonas* were exclusive in siderophore and arylsulfatase producers. In APB group, the relative abundance of *Pseudomonas* was higher in S than in NS.

### 3.6. Evaluation of Nutrient Mobilizing Potential of the Isolates

Screening for phosphate solubilization, siderophore production, and arylsulfatase activity were performed in each group of the isolates (Figure S8). For Ca-PSB, JSC13 provided the maximum phosphate solubilization index (2.20) from tricalcium phosphate, followed by isolates JSC22, JSC10, and JSC5, all belonging to *Burkholderia*. *Burkholderia* sp. JSA1 provided the highest aluminum phosphate solubilization index (4.63) among the Al-PSB. It was followed by *Burkholderia* spp. (JSA12 and JSA5) and *Paraburkholderia* sp. JSA10. Moreover, *Burkholderia* spp. (JSF10, JSF15, and JSF12) and *Pseudomonas* sp. JSF8 were siderophore producers; isolate JSF10 had the highest index among SPB. In the APB group, isolates JSS3, JSS4, and JSS11, classified as *Pseudomonas*, had the maximum arylsulfatase activity.

**Figure 8:**
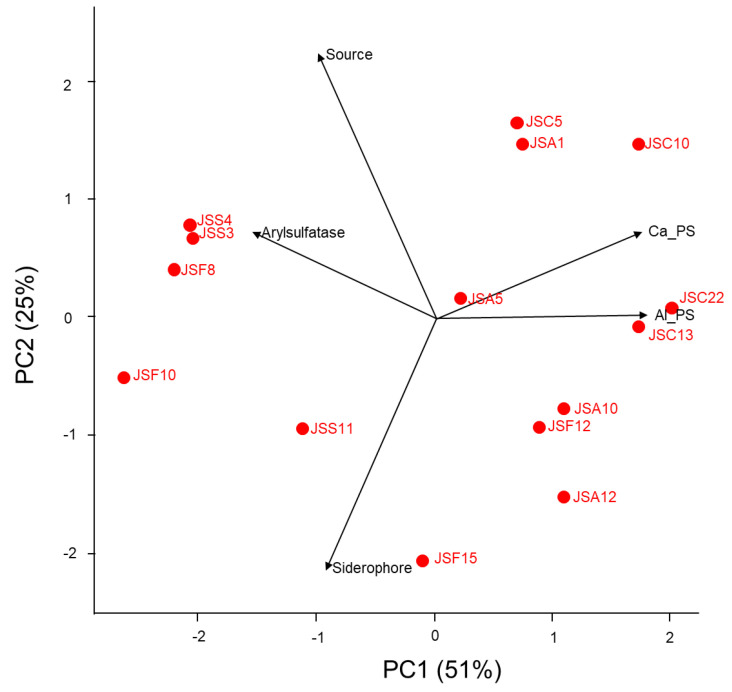
Principal component analysis (PCA) of isolates for multiple nutrient-mobilizing traits. Ca_PS: P solubilization from tricalcium phosphate; Al_PS: P solubilization from aluminum phosphate; Siderophore: production of siderophore; Arylsulfatase: with arylsulfatase activity; Source: isolation source from non-sulfur- or sulfur-applied rhizosphere.

The top-performing isolates in terms of respective PGP abilities were used to determine an isolate that has multiple capabilities for traits on nutrient mobilization (Table S6, Figure 8). The associations between the isolates and nutrient-mobilizing traits were visualized using a principal component analysis (PCA) plot. The axes PC1 (51%) and PC2 (25%), in total 76%, explained the variance of the screened isolates with their abilities. It was found out that isolates *Burkholderia* spp. JSC22 and JSC13 from NS showed a positive relationship with aluminum phosphate solubilization. On the other hand, the *Burkholderia* sp. JSC10 isolated from the S-applied rhizosphere showed positive correlation with calcium phosphate solubilization. The isolates (*Pseudomonas* spp. JSS3 and JSS4) from S application had strong arylsulfatase activity while *Burkholderia* sp. JSF15 isolated from NS had positive association with siderophore production. Isolates with strong positive correlation with phosphate solubilization from tricalcium phosphate and arylsulfatase activity were obtained from S-applied soil. In contrast, the isolates associated with siderophore and aluminum phosphate solubilization were obtained from NS. Finally, the isolate Burkhold eria sp. JSA5 from S-applied soil was capable to the screened multiple nutrient-mobilizing traits among the isolates.

## 4. Discussion

Plants can change the soil microbiota by excreting bioactive molecules in the rhizosphere [45] and may have a specific and promoting effect on microbial populations [46]. Rhizodeposits are rich in carbohydrates and nitrogen compounds that can support a denser microbial community as compared to rootless soil [47,48]. As observed, there is a significant microbial structure shift in soybean cultivation (Figure 2B). The root exudates from soybean can serve as nutrients as well as signaling molecules for recruiting soil microbes towards the rhizosphere. The abundance of phyla *Nitrospirae* and *Acidobacteria* increased with soybean cultivation. Similarly, Igiehon and colleagues observed these two phyla as core bacteria in soybean rhizospheric soil [49]. These two phyla might include potential beneficial bacterial species such as *Nitrospirae* that can promote the nitrogen cycle and increase the availability and utilization of nitrogen in plant [50] and duckweed growth-promoting ability of strains under *Acidobacteria* [51].

In connection, fertilizer regime also influences the soil microbial community [13,14]. Changes in individual bacterial phyla were observed, such as the abundance of Acidobacteria, including the classes Acidobacteria and Solibacteres, which increased with S application. This phylum might have a role in peatland S cycling and includes species-harboring genes for dissimilatory sulfite or sulfate respiration [52]. This is in parallel with the findings that S fertilizer in cadmium- and arsenic-contaminated rice paddy soils promoted the proliferation of S-reducing bacteria [53,54]. As the availability of specific nutrients affect the microbe residing in it, there is no substantial shift in microbial community with the application of S in the rhizosphere. It might be that there are other S sources such as root exudates from soybean for the bacterial metabolism and, accordingly, no drastic changes in the microbial community. Thus, S, as a nutrient for soil microbes, was not the major microbiome-influencing factor in the soybean rhizosphere in this study.

Interestingly, application of S increased organic acids, specifically malic acid, excreted by soybean planted in available S- and phosphorus-deficient soil. S application had no drastic effect on soybean growth in the present study. Soybean seeds contain up to 0.46% (*w*/*w*) of S [55] and might be enough source for up to the six-week-old plants. Furthermore, the P content of the plants did not differ significantly between treatments (Figure S3). This contrasts with the finding that plant P content was also increased as a consequence of increased organic acid secretion brought upon by S application to soybean with tricalcium phosphate as the sole P source [11]. The test soil used in the current study was Andosol, which is commonly characterized as possessing an abundance of active Al and Fe [56]; thus, the dominant form of P might be bound to Al or Fe considering the high P adsorption capacity of the soil. Aside from the P form, the citric acid excreted from the roots may have high efficiency in solubilizing P with S treatment [11,12]. In the present study, the citric acid excreted from S-applied soybean was likely higher but not considerable as compared to the NS-applied soybean. The disparity might be due to Fe not being supplemented in this study. Single or combined Fe and S deficiency in tomato plants showed increased citric acid content in the roots while the other organic acids such as succinic, malic, and fumaric acids were decreased [57]. Moreover, the citrate synthase activity was significantly higher in combined Fe and S deficiency. The current study was limited to root-secreted organic acid concentrations; it might be that citric acid was accumulated inside the plants rather than excreted as response to the deficiency. On the other hand, secretion of malic acid from soybean roots improved with S application. Malate is considered a poor mobilizer of plant nutrients in the rhizosphere [58]. Furthermore, there was no significant malic acid effect in Al speciation or formation of complexes [59]. This indicates that the role of malic acid upon S application did not have a direct influence on soybean P solubilization in this study.

Malic acid significantly increased by S application may play a role in recruiting beneficial bacteria in soybean (Figure 1). Organic acids stimulate a positive chemotactic bacterial response [60]. Studies have shown that the function of root exudate malic acid as chemoattractant signals beneficial bacteria in rice [61,62]. In this study, the abundance of *Polaromonas* was preferential in S application (Figure 4) and showed a significant positive relationship with malic acid (Figure 5). This genus is known to exhibit sulfatase activity, which can aid in plant sulfur supply [63] and has been observed promote plant growth in sugar beet [64]. Moreover, the population of *Pseudomonas* isolated from S-applied soil with arylsulfatase activity was higher compared to the NS condition (Figure 7). Similarly, a high percentage of arylsulfatase-producing bacteria with *Pseudomonas* as the dominant genus were observed in wheat treated with elemental sulfur [39]. *Pseudomonas aeruginosa* possessed specific chemoreceptor PA2652, which is involved in the response to malate but not to other organic acids such as succinate, acetate, or citrate [65]. It was also noted that chemotaxis of *Pseudomonas fluorescens* to L-malate was involved in root colonization of tomato [66]. Contrarily, the abundance of genus *Mycobacterium* had a negative association with malic acid and was found to be favored in the NS rhizosphere. Likewise, *Acidibacter* was favored in NS, which is a gammaproteobacterium capable of reducing ferric iron [67]. Siderophore producers showed lesser percentage in S-applied soil as well as a negative association with malic acid. It follows that the function of malic acid as a repellant to bacteria associated with iron mobilizing must be confirmed. Nevertheless, the biomolecules secreted in plant roots are complex, and organic acid is just one component.

Diverse bacterial communities colonizing plants, i.e., rhizospheres, have various functions that can be beneficial to plants, such as nutrient mobilization. These rhizobacteria, referred to as PGPB, can provide access to nutrients otherwise unavailable to roots. Application of S to wheat modulates the abundance of bacteria with nutrient-mobilizing capacity such as arylsulfatase activity, phosphorus solubilization, and siderophore production [39]. Isolates with a strong positive correlation to phosphate solubilization from tricalcium phosphate and arylsulfatase activity were obtained from S-applied soil. In contrast, the isolates associated with siderophore and aluminum phosphate solubilization were seen in the NS condition. Lastly, the isolate *Burkholderia* sp. JSA5, displaying multiple nutrient-mobilizing traits, was seen in S-applied soil (Table S6). *Burkholderia* is a common phosphate solubilizer and strains under this genus isolated from paddy fields in Japan can solubilize phosphate from various inorganic P sources such as tricalcium, aluminum, or iron phosphates [68]. Additionally, the *Burkholderia* strains inhabiting white lupin cluster roots can produce a large amount of siderophores, which can help in the colonization of the species [69]. This is the first study to describe *Burkholderia’s* arylsulfatase production abilities. The application of S to soybean affects the abundance of nutrient-mobilizing bacteria, subsequently harnessing these beneficial bacteria for sustainable crop production. However, the effect of these promising bacteria for nutrient mobilization of P, Fe, and S on soybean remains to be confirmed.

## 5. Conclusions

S application affected the bacterial community structure of the soybean rhizosphere in Andosol, suggesting a contribution to plant condition change, such as the increase in organic acid secretion. The shift in microbiota as well as isolated strains from S-fertilized soil showed ability related to PGPB activity, and that isolated bacteria have the potential to be harnessed for crop productivity using P, Fe, and S nutrient mobilization.

## Data Availability

The accession number for each gene used in this manuscript are deposited in the DDBJ. The physiological characterization data are included in the manuscript (the raw data only if it is necessary, the authors agree to share with the reviewers).

## Supplementary Materials

The following supporting information can be downloaded at: https://www.mdpi.com/article/10.3390/microorganisms11051193/s1. Figure S1: Arylsulfatase-producing bacteria exhibiting varying intensities of blue color colonies grown in M9 minimal medium with chromogenic 5-bromo-4-chloro-3-indolyl sulfate. Figure S2: Shoot and root dry weights of soybean cultivar Enrei after six weeks after sowing under non-sulfur (NS) and sulfur (S) application treatments. Significant difference between the treatments determined by T-test (*** *p* < 0.001; ** *p* < 0.01; * *p* < 0.05). The error bar indicates the standard error of five replications. Figure S3: P content of soybean cultivar Enrei after six weeks after sowing under non-sulfur (NS) and sulfur (S) application treatments. Significant difference between the treatments determined using t-test (*** *p* < 0.001; ** *p* < 0.01; * *p* < 0.05). The error bar indicates the standard error of four replications. Figure S4: Rarefaction curve for the soil samples uncultivated Bulk (S) or cultivated (R) with soybean “Enrei” under non-sulfur (NS) or sulfur (S) applications based on observed OTUs. Figure S5: Relative abundance of phyla across the samples (A), soybean cultivation (B), and S application (C). RS: with soybean cultivation and sulfur application; RNS: with soybean cultivation and non-sulfur application; SS: without soybean cultivation and sulfur application; SNS: without soybean cultivation and non-sulfur application; Rhizosphere: with soybean cultivation; Bulk: without soybean cultivation; S: sulfur application; NS: non-sulfur application. Figure S6: Percentage to total bacterial population of PSB, SPB, and APB residing in the rhizosphere of non-sulfur (NS) and sulfur (S) applied soybean. APB: arylsulfatase producing bacteria; SPB: siderophore producing bacteria; Al-PSB: bacteria that can solubilize phosphorus from aluminum phosphate; Ca-PSB: bacteria that can solubilize phosphorus from tricalcium phosphate. Figure S7: Redundancy analysis plot of the relationships between plant traits and population of nutrient-mobilizing bacteria residing in the soybean rhizosphere with NS and S treatments. SPcontent: shoot P content; APB: arylsulfatase-producing bacteria; AlPSB: aluminum phosphate-solubilizing bacteria; CaPSB: tricalcium phosphate-solubilizing bacteria; SPB: siderophore-producing bacteria. Figure S8: Indices of isolates for tricalcium (Ca-P) phosphate solubilization (A), aluminum (Al-P) phosphate solubilization (B), and siderophore production (C). S: sulfur application; NS: non-sulfur application. Significant difference between the isolates determined using Tukey’s test (*p* < 0.05). The error bar indicates the standard error of three replications. Table S1: Physicochemical properties of forest soil used in the study. Table S2: Chemical reagents as nutrients amended in the soil for soybean experiment. Table S3: PERMANOVA on the significance of the influence of soybean cultivation and sulfur fertilization on soil microbial community structure. Table S4: Spearman’s rank correlation coefficients of the abundance of microbial community and organic acids from soybean under sulfur applications. Table S5: Characteristics and identity of cultivable bacteria from rhizospheres of NS- and S-applied soybean. Table S6: Characterization of top fifteen isolates with multiple nutrients mobilizing traits.
