# Supplementary material for: The Application of Sulfur Influences Microbiome of Soybean Rhizosphere and Nutrient-Mobilizing Bacteria in Andosol
Source: Microorganisms. 2023 May 3;11(5):1193. doi: 10.3390/microorganisms11051193 (PMC10223564; doi:10.3390/microorganisms11051193)
Supplement: Supplementary file 1 [file microorganisms-11-01193-s001.zip › Supplementary Figures.pdf]

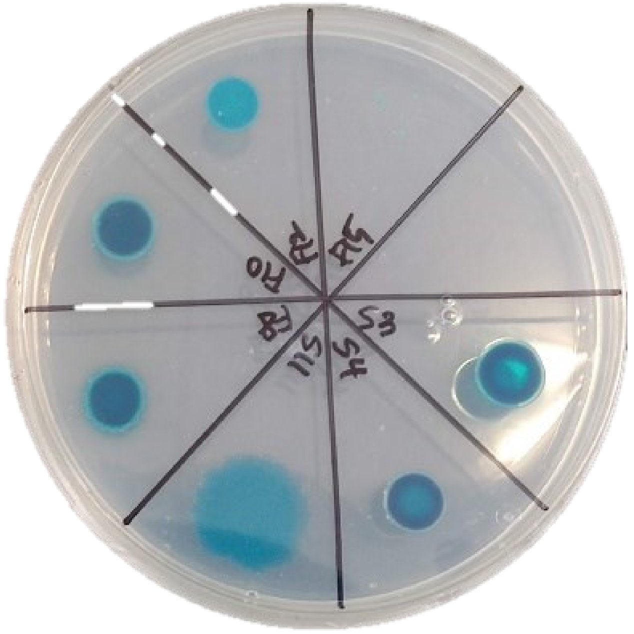

Figure S1. Arylsulfatase-producing bacteria exhibiting varying intensities of blue color colonies grown in M9 minimal medium with chromogenic 5-bromo-4-chloro-3-indolyl sulfate.

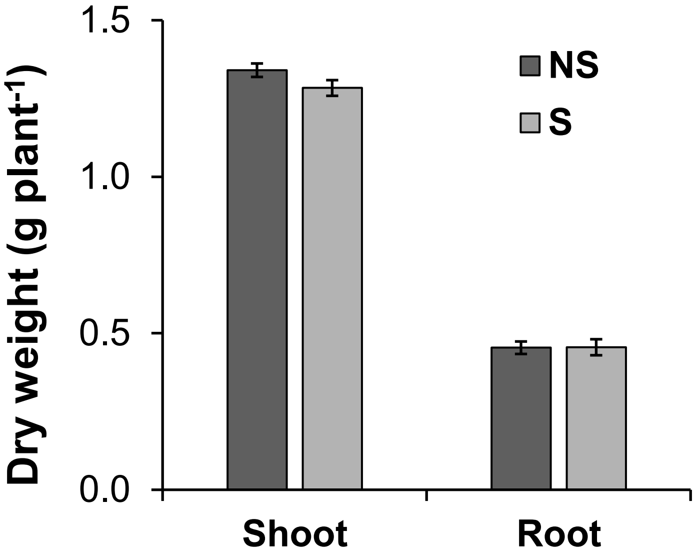

Figure S2. Shoot and root dry weights of soybean cultivar Enrei after six weeks after sowing under non-sulfur (NS) and sulfur (S) application treatments.

Significant difference between the treatments determined by *t*-test . The error bar indicates the standard error of five replications.

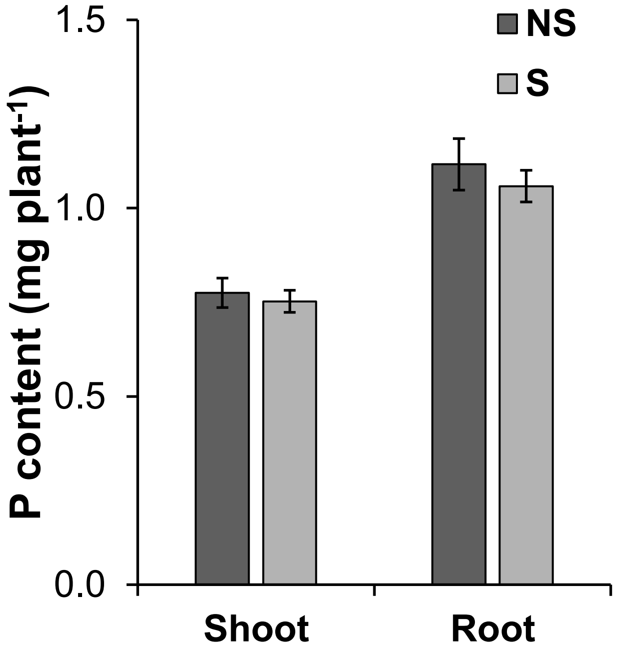

Figure S3. P content of soybean cultivar Enrei after six weeks after sowing under non-sulfur (NS) and sulfur (S) application treatments.

Significant difference between the treatments determined by *t*-test. The error bar indicates the standard error of four replications.

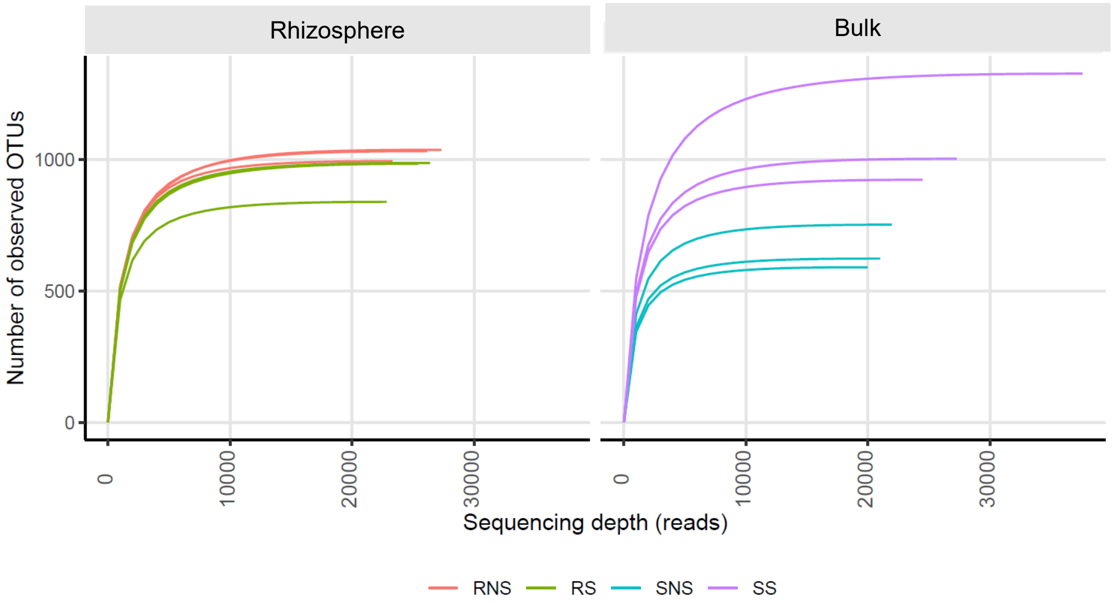

Figure S4. Rarefaction curve for the soil samples uncultivated Bulk (S) or cultivated (R) with soybean 'Enrei' under non-sulfur (NS) or sulfur (S) applications based on observed OTUs.

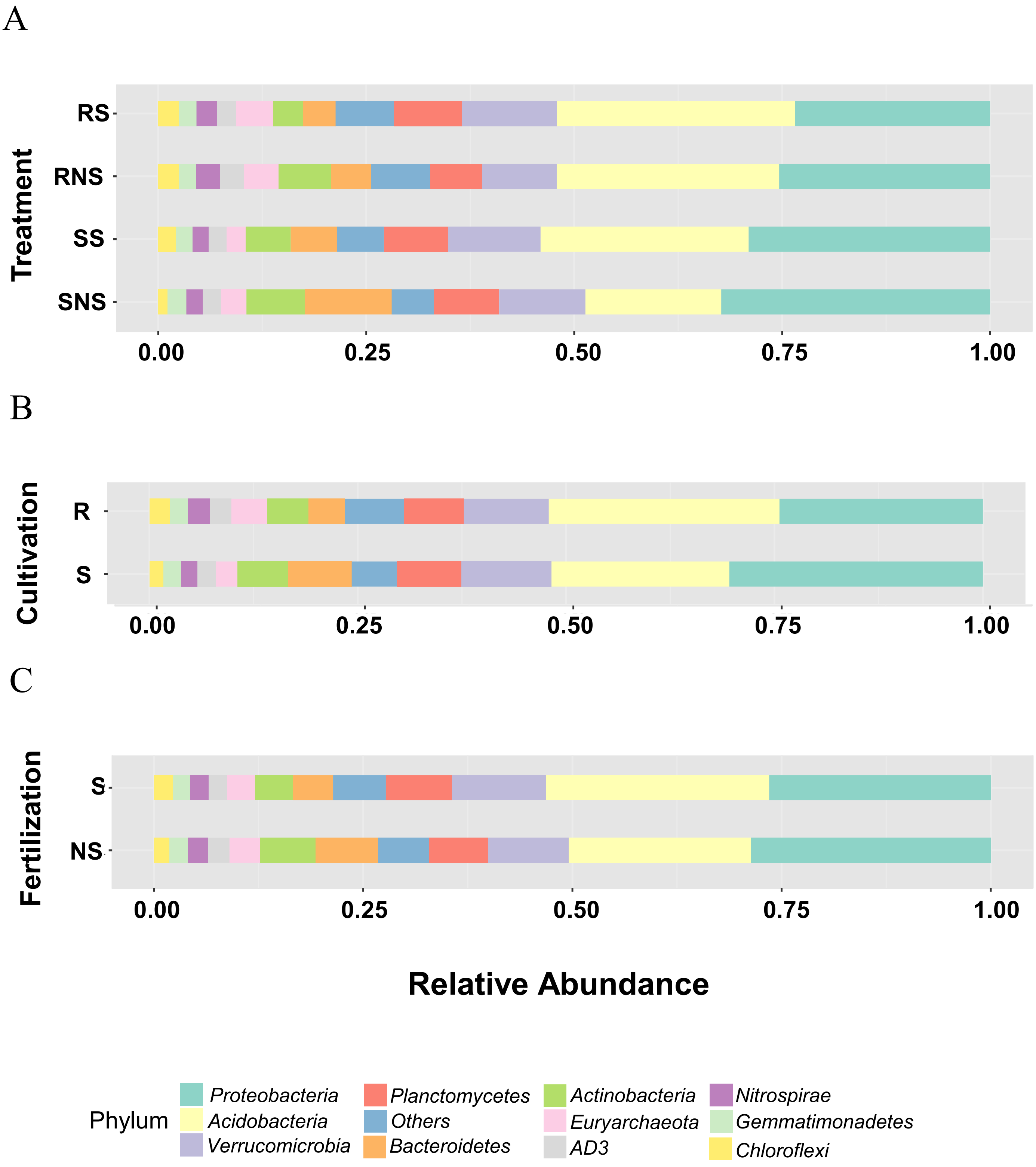

Figure S5. Relative abundance of phyla across the samples (A), soybean cultivation (B), and S application (C).
RS: with soybean cultivation and sulfur application; RNS: with soybean cultivation and non-sulfur application; SS: without soybean cultivation and sulfur application; SNS: without soybean cultivation and non-sulfur application; Rhizosphere: with soybean cultivation; Bulk: without soybean cultivation; S: sulfur application; NS: non-sulfur application

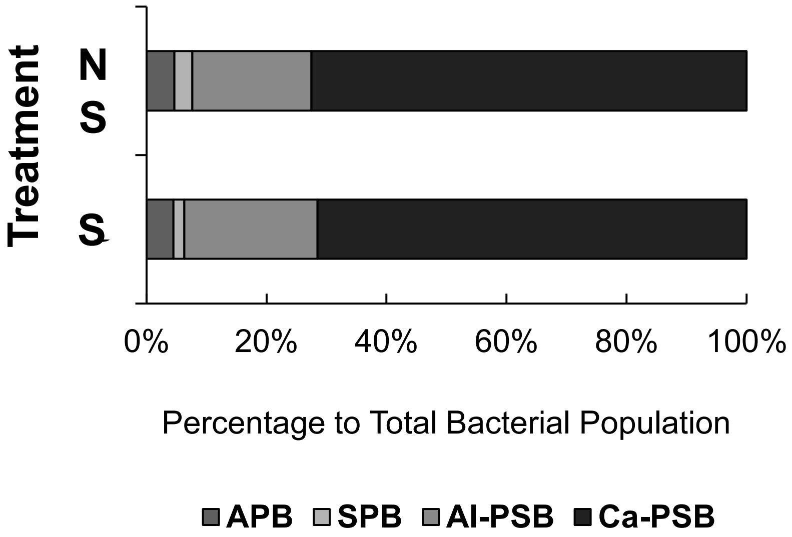

Figure S6. Percentage to total bacterial population of PSB, SPB, and APB residing in the rhizosphere of non-sulfur (NS) and sulfur (S) applied soybean.

APB: arylsulfatase producing bacteria; SPB: siderophore producing bacteria; Al-PSB: bacteria that can solubilize phosphorus from aluminum phosphate; Ca-PSB: bacteria that can solubilize phosphorus from tricalcium phosphate

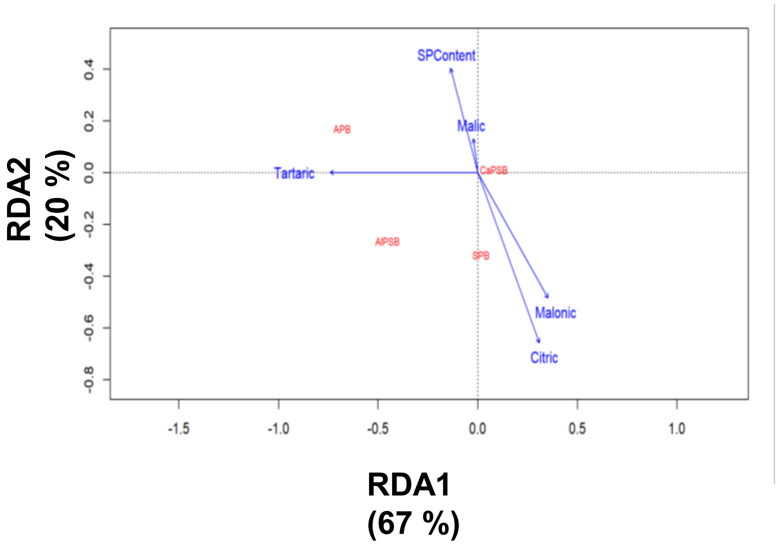

Figure S7. Redundancy analysis plot of the relationships of the plant traits and population of nutrient mobilizing bacteria residing in the soybean rhizosphere with NS and S treatments.

SPcontent: shoot P content; APB: arylsulfatase producing bacteria; AlPSB: aluminum phosphate solubilizing bacteria; CaPSB: tricalcium phosphate solubilizing bacteria; SPB: siderophore producing bacteria

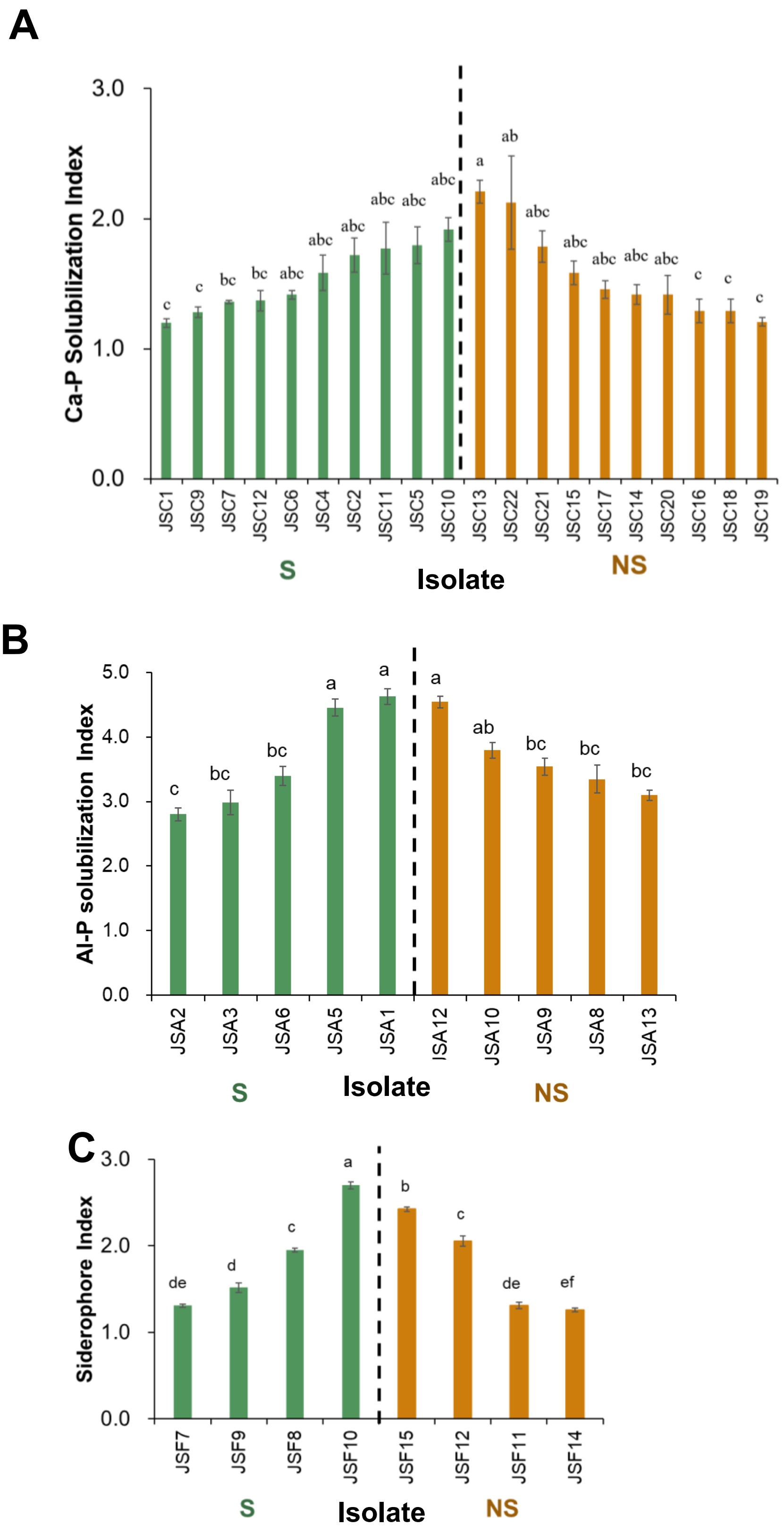

Figure S8. Indices of isolates for tricalcium (Ca-P) phosphate solubilization (A), aluminum (Al-P) phosphate solubilization (B), and siderophore production (C).

S: sulfur application; NS: non-sulfur application. Significant difference between the isolates determined by Tukey's test ($p < 0.05$). The error bar indicates the standard error of three replications.
