# Supplementary material for: The Application of Sulfur Influences Microbiome of Soybean Rhizosphere and Nutrient-Mobilizing Bacteria in Andosol
Source: Microorganisms. 2023 May 3;11(5):1193. doi: 10.3390/microorganisms11051193 (PMC10223564; doi:10.3390/microorganisms11051193)
Supplement: Supplementary file 1 [file microorganisms-11-01193-s001.zip › Supplementary Tables.pdf]

Table S1. Physicochemical properties of forest soil used in the study.

pH	Total C (g C kg^{-1})	Total N (g N kg^{-1})	Available S* (mg S kg^{-1})	Soluble P* (mg P_2O_5 100 g^{-1})	P adsorption capacity* (mg P_2O_5 100 g^{-1})	Feo (g kg^{-1})	Fed (g kg^{-1})	Alo (g kg^{-1})	Ald (g kg^{-1})
5.2 ± 0.1	76.9 ± 1.9	4.9 ± 0.1	21.3 ± 2.6	2.3 ± 0.6	2756.6 ± 17.7	29.2 ± 0.4	43.6 ± 1.0	71.1 ± 0.3	17.4 ± 0.8

*Lifted from Sugiura et al., 2022

Fe_o and Al_o were Fe and Al extractable from acid oxalate method, respectively.

Fe_d and Al_d were Fe and Al extractable from dithionite-citrate reduction method, respectively.

Table S2. Chemical reagents as nutrients amended in the soil for soybean pot experiment.

Nutrient	Sulfur treatment (S)		Non-sulfur treatment (NS)	
	**Chemical Reagent**	**(mg/kg)**	**Chemical Reagent**	**(mg/kg)**
K	K_2SO_4	170.0	KCl	145.5
Mg	$MgSO_4 \cdot 7H_2O$	389.1	$MgCl_2 \cdot 6H_2O$	320.9
N	Urea (CH_4N_2O)	214.4	Urea (CH_4N_2O)	214.4
Cu	$CuCl_2 \cdot 2H_2O$	1.4	$CuCl_2 \cdot 2H_2O$	1.4
Mn	$MnCl_2 \cdot 4H_2O$	0.5	$MnCl_2 \cdot 4H_2O$	0.5
Co	$CoCl_2 \cdot 6H_2O$	0.4	$CoCl_2 \cdot 6H_2O$	0.4
B	H_3BO_3	0.5	H_3BO_3	0.5
Mo	$Na_2MnO_4 \cdot 2H_2O$	0.5	$Na_2MnO_4 \cdot 2H_2O$	0.5
Zn	$ZnCl_2$	1.1	$ZnCl_2$	1.1

Table S3. PERMANOVA on the significance of the influence of soybean cultivation and sulfur fertilization on soil microbial community structure.

	**Soybean Cultivation**			**Sulfur Application**		
	**F**	**R^2**	**p**	**F**	**R^2**	**p**
Microbial Community Structure	6.8081	0.4050	<0.002	1.0448	0.0946	<0.318

F values indicate the magnitude of change on the microbial community structure. Significance level at p<0.05.

Table S4. Spearman's rank correlation coefficients of the abundance of microbial community and organic acids from soybean under sulfur applications.

Genus	Malonic	Malic	Tartaric	Citric
Aquicella	0.41	0.57^{+}	0.46	0.46
Arthrobacter	-0.41	-0.53^{+}	-0.32	-0.44
Bacillus	-0.75^{**}	-0.86^{**}	-0.80^{**}	-0.77^{**}
Burkholderia	0.82^{**}	0.92^{**}	0.91^{**}	0.84^{**}
Catenulispora	0.48	0.64^{*}	0.80^{**}	0.49
Chthoniobacter	-0.62^{*}	-0.70^{*}	-0.64^{*}	-0.57^{+}
Dyella	0.72^{**}	0.54^{+}	0.68^{*}	0.78^{**}
Edaphobacter	-0.65^{*}	-0.68^{*}	-0.68^{*}	-0.69^{*}
Flavisolibacter	-0.82^{**}	-0.89^{**}	-0.85^{**}	-0.82^{**}
Ideonella	-0.76^{**}	-0.82^{**}	-0.75^{**}	-0.78^{**}
Labilithrix	0.43	0.63^{*}	0.54^{+}	0.45
Mesorhizobium	0.82^{**}	0.84^{**}	0.95^{**}	0.83^{**}
Mycobacterium	-0.30	-0.54^{+}	-0.34	-0.27
Nitrospira	0.59^{*}	0.63^{*}	0.60^{*}	0.60^{*}
Paraburkholderia	0.80^{**}	0.90^{**}	0.89^{**}	0.78^{**}
Polaromonas	0.39	0.52^{+}	0.46	0.33
Rhizobium	0.53^{+}	0.53^{+}	0.73^{**}	0.54^{+}
Sphingomonas	-0.89^{**}	-0.86^{**}	-0.84^{**}	-0.88^{**}
Streptomyces	0.79^{**}	0.72^{**}	0.85^{**}	0.81^{**}
Undibacterium	0.58^{*}	0.78^{**}	0.66^{*}	0.55^{+}

Significant relationships at ** $p < 0.01$; * $p < 0.05$; + $p < 0.10$.

Table S5. Characteristics and identity of cultivable bacteria from rhizospheres of NS and S applied soybean.

Isolate	Source	Type of Isolation*	% Identity	16S rRNA	Gram Reaction[a]	Cellular morphology[b]	Colony morphology[c]
JSC1	S	Ca-PSB	93.8	*Paraburkholderia* sp.	-	coccobacilli	entire, translucent, convex, moist, punctiform
JSC2	S	Ca-PSB	97.1	*Burkholderia* sp.	-	coccobacilli	entire, cream, convex, moist, punctiform
JSC4	S	Ca-PSB	93.1	*Burkholderia* sp.	-	coccobacilli	entire, cream, convex, moist, punctiform
JSC5+	S	Ca-PSB	94.9	*Burkholderia* sp.	-	coccobacilli	entire, cream, convex, moist, punctiform
JSC6	S	Ca-PSB	97.3	*Paraburkholderia* sp.	-	coccobacilli	entire, cream, convex, shiny, punctiform
JSC7	S	Ca-PSB	94.3	*Paraburkholderia* sp.	-	coccobacilli	entire, cream, convex, moist, punctiform
JSC9	S	Ca-PSB	98.9	*Paraburkholderia* sp.	-	coccobacilli	entire, cream, convex, moist, punctiform
JSC10+	S	Ca-PSB	99.7	*Burkholderia* sp.	-	coccobacilli	entire, cream, convex, moist, punctiform
JSC11	S	Ca-PSB	99.1	*Burkholderia* sp.	-	coccobacilli	entire, cream, convex, moist, punctiform
JSC12	S	Ca-PSB	99.6	*Paraburkholderia* sp.	-	coccobacilli	entire, cream, convex, moist, punctiform
JSC13+	NS	Ca-PSB	99.8	*Burkholderia* sp.	-	coccobacilli	entire, cream, convex, moist, punctiform
JSC14	NS	Ca-PSB	87.2	*Paraburkholderia* sp.	-	coccobacilli	entire, translucent, convex, shiny, punctiform
JSC15	NS	Ca-PSB	95.9	*Burkholderia* sp.	-	coccobacilli	entire, cream, convex, moist, punctiform
JSC16	NS	Ca-PSB	99.8	*Burkholderia* sp.	-	coccobacilli	entire, cream, convex, moist, punctiform
JSC17	NS	Ca-PSB	99.2	*Burkholderia* sp.	-	coccobacilli	entire, cream, convex, moist, punctiform
JSC18	NS	Ca-PSB	87.6	*Paraburkholderia* sp.	-	coccobacilli	entire, cream, convex, moist, punctiform

JSC19	NS	Ca-PSB	97.0	*Paraburkholderia* sp.	-	coccobacilli	entire, cream, convex, moist, punctiform
JSC20	NS	Ca-PSB	97.9	*Paraburkholderia* sp.	-	coccobacilli	entire, iridescent, convex, moist, punctiform
JSC21	NS	Ca-PSB	95.9	*Burkholderia* sp.	-	coccobacilli	entire, cream, convex, moist, punctiform
JSC22^{+}	NS	Ca-PSB	99.9	*Burkholderia* sp.	-	coccobacilli	entire, cream, convex, moist, punctiform
JSA1^{+}	S	Al-PSB	98.9	*Burkholderia* sp.	-	coccobacilli	entire, cream, convex, moist, punctiform
JSA2	S	Al-PSB	99.1	*Paraburkholderia* sp.	-	coccobacilli	entire, cream, convex, moist, punctiform
JSA3	S	Al-PSB	99.8	*Burkholderia* sp.	-	coccobacilli	entire, cream, convex, moist, punctiform
JSA5^{+}	S	Al-PSB	99.9	*Burkholderia* sp.	-	coccobacilli	entire, cream, convex, moist, punctiform
JSA6	S	Al-PSB	98.9	*Paraburkholderia* sp.	-	coccobacilli	entire, cream, convex, moist, punctiform
JSA8	NS	Al-PSB	99.8	*Burkholderia* sp.	-	coccobacilli	entire, opaque, convex, moist, punctiform
JSA9	NS	Al-PSB	99.8	*Burkholderia* sp.	-	coccobacilli	entire, cream, convex, moist, punctiform
JSA10^{+}	NS	Al-PSB	98.6	*Paraburkholderia* sp.	-	coccobacilli	entire, iridescent, convex, shiny, punctiform
JSA12^{+}	NS	Al-PSB	99.9	*Burkholderia* sp.	-	coccobacilli	entire, cream, convex, moist, punctiform
JSA13	NS	Al-PSB	99.1	*Paraburkholderia* sp.	-	coccobacilli	entire, translucent, convex, shiny, punctiform
JSF7	S	SPB	99.9	*Pseudomonas* sp.	-	coccobacilli	entire, translucent, convex, moist, round
JSF8^{+}	S	SPB	92.9	*Pseudomonas* sp.	-	coccobacilli	entire, iridescent, convex, moist, round
JSF9	S	SPB	98.3	*Burkholderia* sp.	-	coccobacilli	entire, translucent, convex, moist, punctiform
JSF10^{+}	S	SPB	99.9	*Burkholderia* sp.	-	coccobacilli	entire, translucent, convex, shiny, punctiform
JSF11	NS	SPB	99.7	*Pseudomonas* sp.	-	coccobacilli	entire, translucent, convex, moist, round

JSF12+	NS	SPB	98.4	*Burkholderia* sp.	-	coccobacilli	entire, opaque, convex, moist, punctiform
JSF14	NS	SPB	83.6	*Pseudomonas* sp.	-	coccobacilli	entire, brown, convex, shiny, punctiform
JSF15+	NS	SPB	97.4	*Burkholderia* sp.	-	coccobacilli	entire, opaque, convex, shiny, punctiform
JSS1	S	APB	95.0	*Pseudomonas* sp.	-	coccobacilli	entire, opaque, convex, moist, round
JSS2	S	APB	90.8	*Burkholderia* sp.	-	coccobacilli	entire, opaque, convex, shiny, punctiform
JSS3+	S	APB	96.0	*Pseudomonas* sp.	-	coccobacilli	entire, opaque, convex, moist, round
JSS4+	S	APB	90.0	*Pseudomonas* sp.	-	coccobacilli	entire, opaque, convex, moist, round
JSS5	S	APB	99.0	*Pseudomonas* sp.	-	coccobacilli	entire, opaque, convex, moist, round
JSS6	S	APB	94.0	*Pseudomonas* sp.	-	coccobacilli	entire, opaque, convex, moist, round
JSS7	NS	APB	100.0	*Pseudomonas* sp.	-	coccobacilli	entire, cream, convex, moist, punctiform
JSS8	NS	APB	96.6	*Burkholderia* sp.	-	coccobacilli	entire, translucent, convex, shiny, punctiform
JSS9	NS	APB	97.2	*Burkholderia* sp.	-	coccobacilli	entire, translucent, convex, moist, punctiform
JSS10	NS	APB	91.0	*Pseudomonas* sp.	-	coccobacilli	entire, translucent, convex, moist, round
JSS11+	NS	APB	93.0	*Pseudomonas* sp.	-	coccobacilli	wavy, translucent, flat, shiny, irregular
JSS12	NS	APB	99.7	*Burkholderia* sp.	-	coccobacilli	entire, cream, convex, moist, punctiform

[*]Ca-PSB: phosphate solubilization from tricalcium phosphate; Al-PSB: phosphate solubilization from aluminum phosphate; SPB: siderophore production based from yellow to orange halo zones; APB: arylsulfatase-producing bacteria observed based on the formation of blue colony

[a]Gregersen's method

[b]Observed in oil immersion objective

[c]Grown on Luria-Bertani agar and observed after 24 hours

[+]Isolates selected for multiple nutrient mobilization traits

Table S6. Characterization of top fifteen isolates with multiple nutrients mobilizing traits.

Isolate	Source	Bacterial Identity	Ca-PS	Al-PS	Siderophore	Arylsulfatase
JSC5	S	*Burkholderia* sp.	2.39±0.06	3.04±0.03	1.02±0.02	+
JSC10	S	*Burkholderia* sp.	2.55±0.07	3.79±0.09	1.00±0.00	+
JSC13	NS	*Burkholderia* sp.	2.90±0.08	3.71±0.09	1.49±0.02	+
JSC22	NS	*Burkholderia* sp.	2.38±0.04	3.31±0.08	1.00±0.00	+
JSA1	S	*Burkholderia* sp.	2.74±0.04	4.42±0.08	1.51±0.02	++
JSA5	S	*Burkholderia* sp.	2.00±0.06	4.06±0.05	2.19±0.04	+
JSA10	NS	*Paraburkholderia* sp.	1.88±0.16	2.94±0.10	1.58±0.15	+
JSA12	NS	*Burkholderia* sp.	2.50±0.03	4.27±0.09	2.63±0.06	+
JSF8	S	*Pseudomonas* sp.	1.11±0.05	1.00±0.00	1.95±0.02	+
JSF10	S	*Burkholderia* sp.	1.06±0.05	1.00±0.00	2.70±0.04	+
JSF12	NS	*Burkholderia* sp.	2.25±0.03	3.64±0.06	2.06±0.06	+
JSF15	NS	*Burkholderia* sp.	1.00±0.00	2.78±0.04	2.42±0.02	+
JSS3	S	*Pseudomonas* sp.	1.15±0.01	1.00±0.00	1,69±0.04	++
JSS4	S	*Pseudomonas* sp.	1.06±0.05	1.00±0.00	1.59±0.02	++
JSS11	NS	*Pseudomonas* sp.	1.72±0.24	1.32±0.01	2.13±0.09	++

Ca-PS: phosphate solubilization index from tricalcium phosphate; Al-PS: phosphate solubilization index from aluminum phosphate; Siderophore: siderophore production index based from yellow to orange halo zones; Arylsulfatase: observed based on the formation of blue colony
